# Murine Progeria Model Exhibits Delayed Fracture Healing With Senescent Phenotype and Dysregulated Immune Response

**DOI:** 10.1002/jor.70193

**Published:** 2026-04-04

**Authors:** Victoria R. Duke, Marc J. Philippon, Dane R. G. Lind, Herbert Kasler, Kohei Yamaura, Matt Huard, Molly Czachor, Justin Hollenbeck, Justin Brown, Alex Garcia, Jacob D. Matityahu, Naomasa Fukase, Ralph S. Marcucio, Anna‐Laura Nelson, William S. Hambright, Dustin M. Snapper, Johnny Huard, Chelsea S. Bahney

**Affiliations:** ^1^ Steadman Philippon Research Institute Vail Colorado USA; ^2^ Buck Institute for Research on Aging Novato California USA; ^3^ Department of Orthopaedic Surgery Kobe University Graduate School of Medicine Kobe Japan; ^4^ University of California San Francisco California USA

**Keywords:** animal model, delayed union, fracture repair, immunophenotyping, non‐union, progeria

## Abstract

An estimated 189 million bone fractures occurred in 2019 making it one of the most globally prevalent injuries. Delayed union or nonunion occurs in up to 15% of normal fractures with higher rates in aged individuals. Preclinical testing supports the translation of novel strategies to promote improved fracture repair, but there is a paucity of small animal models that recapitulate delayed fracture healing. Here, we evaluated the *Zmpste24* ^−^^/^^−^ (Z24^−^^/^^−^) murine model of Hutchinson‐Gilford progeria syndrome as a model of delayed fracture healing. Leveraging the previously characterized Z24^−^^/^^−^ phenotype of genomic instability, epigenetic changes, and fragility, we hypothesize that progeria mice will present with significantly delayed fracture healing relative to age‐matched wild type (WT) controls. Mice received intramedullary‐fixed tibia fractures with healing and immunosenescence evaluated throughout repair. Z24^−^^/^^−^ mice demonstrated significantly delayed healing with smaller fracture calli containing more cartilage and less bone relative to WT mice. The fracture healing phenotype of the Z24^−^^/^^−^ phenocopied naturally aged mice with increased systemic senescence noted in animals relative to adult WT. Unlike naturally aged mice, Z24^−^^/^^−^ also presented with frail bones. Z24^−^^/^^−^ showed a dysregulated immune composition, with decreased lymphopoiesis, increased myelopoiesis and neutrophil accumulation. Aspects of the macrophage phenotype in Z24^−^^/^^−^ reflected changes in natural aging, but with different systemic T cell responses. Given the Z24^−^^/^^−^ progeria mouse model demonstrates the delayed fracture healing phenotype of naturally aged animal at 3 rather than 20 months of age, we suggest this model provides an accelerated model of age‐related delayed fracture healing.

## Graphical Abstract

This study explores whether a murine model of progeria has a delayed fracture healing phenotype that resembles chronological aging but with an accelerated timeline that could make it a valuable research tool.

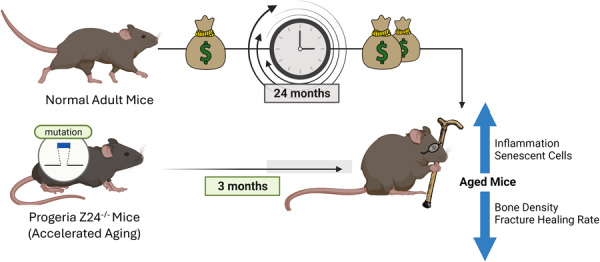

## 1 Introduction

In 2019 the Lancet Global Burden of Disease estimated 189 million fractures occurred annually, an increase of 33.4% since 1990 [1]. While many fractures heal completely without incident, there remains a high prevalence of fractures with impaired healing, with the highest rates found in femoral (13.6%) and tibial (11.7%) shaft fractures [2, 3]. While there remains no single consensus for the clinical definitions of delayed and nonunion, generally, delayed union may be considered for long bone fractures with limited evidence of healing between 3 and 6 months and nonunion is diagnosed following the failure of a fracture to heal at 9 months [4, 5]. Delayed union and nonunion fractures commonly require several surgical procedures to achieve healing significantly extending the treatment period and contributing to lengthy patient disability, persistent pain, and increased healthcare costs [2, 6].

The incidence rate for osteoporotic/osteopenic fractures is significantly increased in the elderly population and contributes to morbidity and mortality in aged individuals [7]. Further, the underlying physiological landscape characterized by chronic low‐grade inflammation is known to disrupt proper bone healing [8]. Given that in the next 30 years the global population of individuals over the age of 65 is projected to double, reaching 1.5 billion by the year 2050 [9], it is critical to develop therapeutics that promote the regeneration of bone after injury to meet the medical challenges faced by an aging population.

One barrier to discovering novel regenerative therapeutics to address delayed healing in the aging population is an unmet need for practical preclinical models. Naturally aged mice have been effectively studied and used to reveal important biological mechanisms underlying delayed fracture healing, most prominently immune and osteoanabolic dysregulation [10, 11, 12]. However, the NIH standard for age‐related research in mice is 24 months, a constraint that creates a substantial time and monetary burden for effectively screening therapies associated with age‐related decline in bone healing. Alternatively, preclinical models that induce diabetes mellitus or rheumatoid arthritis have proven useful in modeling impaired bone union in the presence of chronic inflammation [13, 14, 15]. While these models are clinically relevant to the targeted disease, they often exhibit severely dysregulated immune responses that are absent from the more subtle pathology of fracture healing in the general aged population. Additionally, patients at risk of delayed union commonly present with a frailty phenotype established to negatively influence bone regeneration, including osteoporosis and sarcopenia, that existing fracture models do not capture. As such, there is an unmet need to establish additional preclinical models of delayed fracture healing.

Hutchinson‐Gilford progeria syndrome (HGPS) is a systemic disease of accelerated aging caused by a mutation in the nuclear protein Lamin A [10]. The disrupted nuclear lamin structure alters gene expression and genomic stability, resulting in biological changes observed in natural aging, including DNA damage, cellular senescence, cytoskeletal stiffness, and stem cell exhaustion [11, 12, 16, 17]. Of the various murine models of HGPS established to research age‐related diseases, genetic deletion of *Zmpste24 –* a metalloprotease essential for processing prelamin A into mature lamin A – recapitulates the premature aging phenotype with accompanying musculoskeletal deficits reflective of frail individuals [18, 19, 20]. Within 3–4 months of age, the *Zmpste24^−/−^* (Z24^−^^/^^−^) mouse model exhibits musculoskeletal deficiencies, including reduced myogenic and osteogenic stem cell proliferation and differentiation, bone density loss, sarcopenia, weight loss, osteoporosis and osteoarthritis [11, 21]. Further, frail individuals have also been shown to have reduced circulating osteoprogenitors and reduced lamin A, providing evidence that the Z24^−^^/^^−^ mouse model may have translational relevance to delayed bone regeneration in the context of aged fractures [22].

The primary objective of this study is to evaluate the Z24^−^^/^^−^ progeria mouse as a potential model of delayed fracture healing. We hypothesize that Z24^−^^/^^−^ mice will present with delayed fracture healing when compared to age‐matched wild‐type (WT) controls and exhibit both increased senescent cell burden and a dysregulated immune response. We included a smaller subset of naturally aged WT mice to benchmark the fracture healing phenotype of Z24^−^^/^^−^ mice.

## 2 Methods

### 2.1 Animal Model and Welfare

A Material Transfer Agreement (MTA) was executed with the Universidad de Oviedo and Dr. Carlos Lopez Otin for the use of the *Zmpste24* mice (Recipient Scientists: Drs Johnny Huard and Chelsea Bahney). Z24^−^^/^^−^ progeria mice were generated as previously described through a homozygous deletion of *Zmpste24* on the C57Bl6/J background [11]. For the present study, Z24^−^^/^^−^ mice were created by crossing two heterozygous Z24^−^^/+^ mice and genotyping the progeny for the homozygous deletion. Congenic Z24^+/+^ littermates were used as WT controls. Genotyping was performed using a three‐primer mix (Table 1). WT mice were identified by a DNA band at 520 base pairs and Z24^−^^/^^−^ mice were identified by a DNA band at 303 base pairs. Aged C57Bl6/J mice (18 months) were obtained from Jackson Lab (strain #00064) and housed in‐house for approximately 4 additional weeks for acclimation.

**Table 1:** Mouse primer sequences.

Gene	Forward	Reverse
*Zmpste24*	GCTGGCCTTGTTGCTGGAAT	Wild type: GCTTCCTCCCTGAGCCAACC Knock out: CTTCCGGAGCGGATCTCAAA
*Il*‐*1β*	GTGTGGATCCCAAGCAATAC	GTCCTGACCACTGTTGTTTC
*Tnf*‐*α*	GATTATGGCTCAGGGTCCAA	CTCCCTTTGCAGAACTCAGG
*p16*	ATCGTGCGATATTTGCGTTC	TAGCTCTGCTCTTGGGATTG
*p21*	GTCAGGCTGGTCTGCCTCCG	CGGTCCCGTGGACAGTGAGCA
*Gapdh*	TGATGACATCAAGAAGGTGGTGAAG	CCTTGGAGGCCATGTAGGCCAT

All procedures received approval from the Colorado State University Institutional Animal Care and Use Committee (IACUC) and followed NIH guidelines for ethical treatment of animals. Mice were socially housed and allowed to ambulate freely. DietGel® nutritional supplements (ClearH_2_O®, 72085022) were provided to all mice 14 days prior to and post‐surgery until euthanasia to maintain body weight. The mean experimental age for the Z24^−^^/^^−^ progeria mice was 12 weeks, with a range of 10–14 weeks, to ensure adequate welfare of the mice which have median lifespan of only 19–23 weeks with significant health deterioration around 5 months of age [23].

### 2.2 Murine Tibial Fracture and Stabilization Model

Mice were anesthetized using isoflurane inhalation. The right leg was prepared for surgery by shaving the surgical site, then disinfecting with three rounds of 70% alcohol wipes followed by chlorhexidine surgical scrub solution. Sustained release buprenorphine (0.6–1.0 mg/kg) was then administered and mouse eyes were lubricated using artificial tears ointment. All surgeries were performed on a heated operating table using aseptic technique. To create the fracture, a small incision was made along the tibia, and a 23‐gauge needle was used to form a hole at the top of the tibial plateau. A sterilized intramedullary pin was inserted through the hole spanning from the tibial plateau, through the tibial cavity, and into the distal tibia. A Dremel was used to create two small holes in the mid‐shaft of the tibia and pressure was applied to both the proximal and distal ends to generate a full‐thickness tibial fracture as previously described [24]. The incision was closed with 5‐0 Biosyn Sutures (Covidien, 5687) and one surgical skin staple. Bupivacaine hydrochloride (NovaPlus, RL7562) was applied topically for post‐operative pain management and animals were monitored for evidence of pain or suffering for 72 h. Animals were euthanized 3‐, 9‐, 14‐, or 21 days post‐fracture by carbon dioxide (CO_2_) asphyxiation and tissues harvested for analysis.

At the time of fracture, the Z24^−^^/^^−^ mice and congenic Z24^+/+^ littermates (WT controls) were a mean of 12 weeks old with a range of 10–14 weeks. Both male and female mice were used in this study since these animals were breed in‐house and we wished to use littermate controls. The male to female ratio in our study was not equal with female mice accounting for 70%–80% of the included animals. In our previous studies we have not found significant histological, molecular, or functional differences between fracture healing according to sex [25, 26, 27]. Aged mice were fractured between 75 and 80 weeks old (~19 months). Only female mice were used in this study to increase the likelihood of osteopenic bone structure and to increase animal well‐being through social housing.

### 2.3 Micro‐Computed Tomography (μCT) Analysis

Tibiae were collected 21 days post‐fracture after euthanasia to assess bone formation from the injured site through morphometric analysis performed with ex vivo μCT using a Scanco VivaCT 90 μCT scanner (Scanco Medical AG, CH‐8306, Bruttisellen, Switzerland) with the following parameters: 70kVp; filter: 0.5 mm AL; BH:1200 mg HA/ccm; scaling 4096; voxel size: 15.6 µm. Three‐dimensional images were reconstructed using Scanco Medical Software. Quantification of total volume (TV), bone volume (BV), bone mineral density (BMD), trabecular number (Tb.N), and trabecular thickness (Tb.Th) were performed in the defect region.

### 2.4 Histology and Histomorphometry

Fractured tibiae were dissected 14 and 21 days post‐fracture and fixed in 4% paraformaldehyde (Santa Cruz, SC281692) for 24 h at 4°C. Bones were then decalcified by rocking in 19% ethylenediaminetetraacetic acid (Biorad, 1610729) for 14 days at 4°C with solution changes every other day. Following decalcification, the pin was carefully removed from the tibia, which can sometimes cause displacement of the bone ends. Decalcified tibiae were paraffin embedded and whole samples were serially sectioned into 8 µm sections with three sections per slide. Samples were stained with Hall Brundt's Quadruple staining protocol to visualize osseous (red) and cartilaginous (blue) tissue within the fracture callus as previously described [28].

For histomorphometric quantification of the tissue composition in the fracture callus, the first section of every tenth slide was imaged using a Nikon Eclipse Ni‐U microscope with Nikon NIS Basic Research Elements Software (Nikon Instruments Inc., version 4.30) captured at 2X magnification. The fracture callus region of interest for each image was isolated using the “Lasso” tool on Adobe Photoshop (version 23.0.1) to remove non‐callus tissue from each image. The area of each callus tissue (cartilage, bone, and background) was then quantified using the Trainable Weka Segmentation add‐on in Fiji ImageJ (version 1.51.23; NIH), as previously described [29]. The total volume of each tissue type in the fracture callus was approximated by multiplying the tissue area on each slide by the uniform spacing between each slide over the entire length of the fracture callus. The volumes of each tissue type are expressed as percentages of the total fracture callus volume per mouse.

### 2.5 Immunophenotyping

Inguinal lymph nodes, peripheral blood via the tail vein, and tibia bone marrow were harvested between 0 and 14 days post‐fracture. These samples were analyzed using a 31‐color panel spectral flow panel for peripheral blood and lymph nodes and a 30‐color panel spectral flow panel for bone marrow (Supporting Figure 1). Single‐cell suspensions were prepared from mouse tissues as follows: inguinal lymph nodes were placed in 6‐well or 12‐well plates, in 5 mL or 1 mL of ice‐cold RPMI/10% FBS and crushed with a syringe plunger to dissociate the cells. To remove the stroma, both suspensions were then filtered through a 40 µm nylon mesh (Genesee, 25‐375) and pelleted at 600 × g for 6 min. Supernatants were discarded, and the cells were taken up in 3 mL or 1 mL of ACK lysis buffer as previously described [30] and incubated for 3 min at room temperature (RT). Afterward, 10 times the buffer volume of cold PBS was added, and the cells were again pelleted as above. For peripheral blood, approximately 50 µL was collected from the tail vein into 1.4 mL of cold PBS/2 mM EDTA in microcentrifuge tubes, then pelleted for 4 min at 600 × g. After removal of the supernatant, the pellets were taken up in 350 µL ACK lysis buffer and incubated for 3 min at RT. Afterward, 1.2 mL of cold PBS was added, and the cells were again pelleted. For bone marrow, tibias and femurs were dissected from whole legs and crushed using a syringe plunger in 5 mL RPMI/10% FBS. After vigorous resuspension to dissociate the bone marrow, the suspension was filtered through a 40 µm nylon mesh (Genesee, 25‐375), pelleted at 600 × g for 6 min, and lysed with ACK buffer, followed by dilution with 10 volumes of PBS and pelleting of the cells.

For each single‐cell suspension, samples were then suspended in a minimal volume of PBS and counted. 0.5–2.0 × 10^6^ cells were transferred to 1 mL of RPMI/10% fetal bovine serum (FBS), pre‐warmed to 37°C in microcentrifuge tubes. Bafilomycin A1 (ThermoFisher Scientific, 328120001) was then added at 500 nM in DMSO, and the cells were incubated at 37°C for 45 min. After this time, C_12_FDG (Invitrogen, D2893) was added to 30 µM final concentration and the cells were incubated at 37°C for another 45 min. At the end of the C_12_FDG labeling, the cells were pelleted as above, washed once with 1 mL of cold PBS, taken up in 120 mL of PBS/2 mM EDTA with 2X fixable Live/Dead Blue stain (Invitrogen, L23105), transferred to 96‐well V‐bottom plates, and incubated on ice in the dark for 15 min. After live/dead staining, the antibody cocktails were added neat in the noted volumes (Supporting Table 1) along with sufficient FBS to bring the final concentration to 2%, and the samples were incubated for another 30 min on ice in the dark. The samples were then diluted with 150 µL of PBS/2 mM EDTA/2% FBS, pelleted at 600 × g for 5 min, and resuspended in 150 µL of PBS/2 mM EDTA/2% FBS for acquisition. Sample acquisition was performed on a 5‐laser Cytek Aurora spectral flow cytometer (Cytek Biosciences), and spectral unmixing was done using SpectroFlo software (Cytek). Subsequent manual correction of unmixing and gating was done using FlowJo software (BD Biosciences, version 10.1) to identify unique cell populations.

### 2.6 Mechanical Strength Testing

Fractured and contralateral tibiae were collected 21 days post‐fracture, wrapped in phosphate‐buffered saline (PBS)‐soaked paper towels, and stored at 4°C until testing. The proximal and distal ends of the tibiae were potted in a cylindrical mold using polymethyl methacrylate (Fricke Dental) such that the longitudinal axis of the proximal and distal cylindrical molds was aligned to the anatomical axis of the tibia. Torsional testing to failure was performed in a blinded fashion. Using custom fixtures, each specimen was mounted to an Axial Torsion Test Machine (Test Resources, 131AT) instrumented with a 2 Newton‐meter (N‐m) capacity torque load cell. Torsional load was applied at 1 degree/second until failure occurred. Failure torque (N‐m) and torsional stiffness (N‐m/degree) were measured. Failure torque was defined as the maximum force that the bone sustained before fracture, and torsional stiffness was calculated as the slope of the linear portion of the torque‐rotation curve.

### 2.7 Senescence Quantification

Peripheral blood was collected via tail vein pre‐fracture and cardiac puncture 14 days post‐fracture using a syringe coated with anticoagulant citrate‐dextrose solution A (ACD‐A; McKesson, 353996). Peripheral blood mononuclear cells (PBMCs) were isolated using pluriMate® centrifugation tubes pre‐filled with PBMC Spin Medium® (pluriSelect, 440920215) and control rate frozen in CryoStor® (StemCell Technologies, 7930) until analysis. To quantify cellular senescence, C_12_FDG was used to label cells demonstrating elevated senescence‐associated β‐galactosidase activity [31, 32]. PBMC samples were briefly thawed and stained with 500 nM bafilomycin for false positive reduction (Cell Signaling Technology, 54645), 30 µM C_12_FDG (Abcam, ab273642), and DRAQ7 to identify dead cells (NOVUS Biologicals, NBP2‐81126). Samples were run on a Guava® easyCyte™ HT (Guava®) flow cytometer and analyzed using InCyte software (GauvaSoft 3.3).

### 2.8 Gene Expression Analysis

Fractured tibiae (day 9) were harvested and stored in RNAlater (Invitrogen, AM7020) at −20°C until RNA isolation. Fracture calli were carefully dissected from the tibiae/surrounding muscle and all organs were homogenized in Trizol reagent (Invitrogen, 15596026) on ice. Total RNA was extracted from samples according to the manufacturer's protocol. Reverse transcription of RNA into cDNA was performed via the manufacturer's protocol using qScript cDNA synthesis kit (VWR, 95048100). Quantitative RT‐PCR (qRT‐PCR) using PerfeCTa SYBR Green FastMix (VWR, 101414280) was run on the Step‐One Plus Real‐Time PCR system (Applied Biosystems,4376598). Relative gene expression of interleukin 1 beta (*Il‐1β)*, tumor necrosis factor‐alpha (*Tnf‐α)*, and cell‐cycle regulator proteins *p16* and *p21* was calculated by normalizing results to the housekeeping gene (*Gapdh*, ΔCT) (Table 1). Relative gene expression was calculated using the 2^−ΔCT^ method.

### 2.9 Protein Quantification

Whole blood was collected (21 days post‐fracture) and plasma was isolated using ACD‐A coated tubes, followed by centrifugation at 1500 × g for 10 min. Supernatant was collected and stored at −80°C until analysis. Multiplexed chemokine and cytokine analysis was performed using Milliplex Mouse Cytokine/Chemokine Magnetic Bead Panel (EMD Millipore, MCYTOMAG70K10) according to the manufacturer's protocol and read on a Luminex® 100/200 platform using xPonent® software (EMD Millipore Corp, LX200‐XPON‐RUO, version 4.2). All standards and samples were run in duplicate and analyte concentrations were calculated using Belysa® Immunoassay Curve Fitting Software (EMD Millipore, 40‐122, version 3.4). Standard ELISA kits were used to quantify growth differentiation factor 15 (GDF‐15, R&D systems, MGD150) protein levels following the manufacturer's protocol. All standards and samples were run in duplicate using the Infinite® M plex microplate reader (Tecan, 30190085).

### 2.10 Statistical Analysis

Statistical analyses were performed using GraphPad Prism (Version 9.4) and sample size and specific statistical test is indicated within each figure. The non‐parametric Mann‐Whitney U test was used to determine significance between two groups. Flow immunophenotyping data was analyzed using a two‐tailed *t*‐test. Comparisons among multiple groups were performed using one‐way ANOVA followed by Tukey's post‐hoc testing. Time course data was analyzed using a mixed‐effects model followed by Tukey's multiple comparison correction and post‐hoc testing. *p* < 0.05 was used to indicate statistical significance.

## 3 Results

### 3.1 Z24^−^^/^^−^ Mice Exhibit Delayed Fracture Healing and Decreased Torsional Bone Strength Compared to Age‐Matched WT Mice

**Figure 1:**
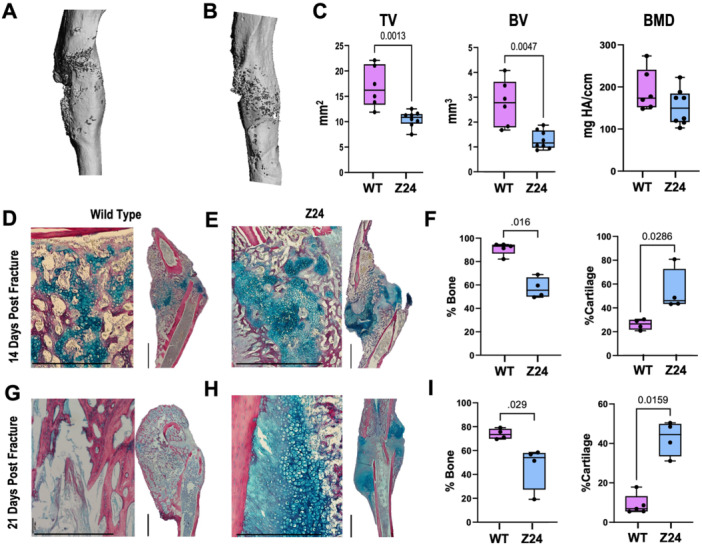
Delayed healing in Z24^−^^/^^−^ mice following fracture compared to age‐matched WT mice. Representative μCT images of fracture callus bone architecture at 21 days post‐fracture in (A) WT and (B) Z24^−^^/^^−^ mice. (C) Bone mineral density (BMD), total volume (TV), and bone volume (BV) 21 days post‐fracture (*n*: WT = 6, Z24^−^^/^^−^ = 8). Significance was defined by *p* ≤ 0.05 determined by Mann‐Whitney *U* test. Hall Brundt's Quadruple stained images of representative mice tibiae fracture calli (D–E) 14 and (G–H) 21 days post‐fracture (Scale bar: 1 mm (left, 10X), 2 mm (right, 2X)). Histomorphometric quantification of bone and combined cartilage and fibrous tissue volumes in tibial fracture callus (F) 14 and (I) 21 days after fracture (*n*: WT = 5, Z24^−^^/^^−^ = 4).

The healing response in the Z24^−^^/^^−^ progeric mice was reduced compared to the WT mice as indicated by the significantly decreased total volume (TV) of the fracture callus (Figure 1A–C, *p* = 0.013). Further, μCT analysis shows that the fracture callus composition was altered with a specifically reduced bone volume (BV, Figure 1C, *p* = 0.0047). BMD within the fracture callus was not significantly decreased in Z24^−^^/^^−^ mice (*p* = 0.2284, Figure 1C).

Histologically, at both 14 (Figure 1D–E) and 21 days (Figure 1G–H) post‐fracture, fracture calli from Z24^−^^/^^−^ mice grossly displayed increased cartilage retention (blue) and decreased bone (red) within the callus compared to that of age‐matched WT mice. Quantitative histomorphometry revealed 37.1% lower bone volume (*p* = 0.029) and 107.0% higher cartilage tissue volume (*p* = 0.0286) in Z24^−^^/^^−^ mice compared to WT mice 14 days post‐fracture (Figure 1F). At 21 days post‐fracture with Z24^−^^/^^−^ mice continued to have a reduction in bone volume (33.7%, *p* = 0.016) and increased in cartilage volume (379.7%, *p* = 0.0159. Figure 1I).

### 3.2 Systemic Dysregulation of the Innate and Adaptive Immune System During the Time Course of Fracture Repair in Z24^−^^/^^−^ Mice

**Figure 2:**
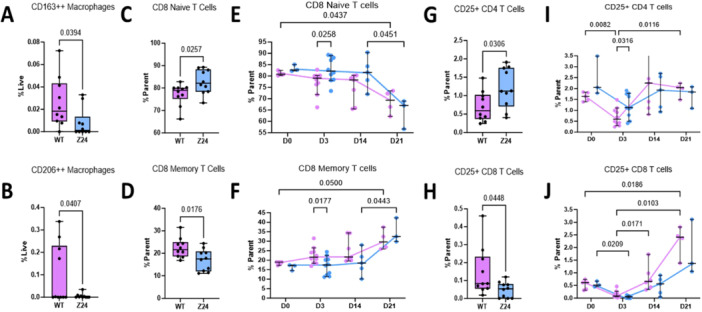
Reduced systemic anti‐inflammatory macrophages and unique T cell repertoire in Z24^−^^*/*^^−^. Spectral flow immunophenotyping analysis of blood collected 3 days post fracture evaluating the quantity of (A) CD163^++^ macrophages, (B) CD206^++^ macrophages, (C) CD8^+^ naïve T cells, (D) CD8^+^ memory T cells, (G) CD25^+^CD4^+^ T cells, and (H) CD25^+^CD8^+^ T cells (*n*: WT = 10, Z24^−^^/^^−^ = 10). *p* ≤ 0.05 determined by two‐tailed T‐test. Time course analysis of Z24^−^^/^^−^ blood collected before fracture and 3‐, 14‐, and 21 days post‐fracture evaluating the quantity of (E) CD8^+^ naïve T cells, (F) CD8^+^ memory T cells, (I) CD25^+^CD4^+^ T cells, and (J) CD25 + CD8^+^ T cells. *p* *< *0.05 determined by Mixed‐effects analysis (two‐way ANOVA) with Tukey multiple comparisons correction test.

Systemic changes to PBMCs collected from peripheral blood were evaluated before injury and at days 3, 14, and 21 post‐fracture through precision immunophenotyping to identify all major myeloid and lymphoid cell types using 30‐channel spectral flow. (Supporting Table 1–2, Supporting Figure 1). At day 3 post‐fracture, Z24^−^^/^^−^ PBMCs contained significantly fewer CD163^++^ (*p* = 0.0394, Figure 2A) and CD206^++^ (*p* = 0.0407, Figure 2B) macrophages compared to the age‐matched WT mice. No significant differences were found in the relative quantities of neutrophils between the Z24^−^^/^^−^ and WT mice (*p* = 0.7044, Supporting Figure 2A).

Pronounced differences were also observed in the adaptive immune system phenotype, specifically related to T cell populations. At day 3 following fracture, the Z24^−^^/^^−^ mice had a higher proportion of CD8^+^ naïve T cells (*p* = 0.0257, Figure 2C) and a smaller proportion of CD8^+^ memory T cells (*p* = 0.0176, Figure 2D). Within both the Z24^−^^/^^−^ and WT mice peripheral blood, the percentage of naïve CD8^+^ T cells stayed relatively constant throughout the endochondral phase of repair, but then demonstrated a relative decrease at day 21 (*p* = 0.0451, Figure 2E). The percentage of CD8^+^ memory T cells similarly stayed constant through day 14, but then increased 21 days post fracture, concomitant with the decrease in naïve CD8^+^ T cells (*p* = 0.0443, Figure 2F).

At day 3, we also found a higher number of activated CD25^+^CD4^+^ T helper cells in the Z24^−^^/^^−^ mice (*p* = 0.0306, Figure 2G) but a smaller proportion of activated CD25^+^CD8^+^ cytotoxic T cells (*p* = 0.0448, Figure 5H). While activated CD25^+^CD8^+^ cytotoxic T cells reached their lowest levels in the blood in both mice genotypes at day 3 (*p* = 0.0209), they rose steadily in quantity to significantly accumulate at day 21 (Figure 2J). Z24^−^^/^^−^ and WT mice displayed similar quantities and subsets of B cells in circulation at all time points (*p* = 0.5119, Supporting Figure 2B).

### 3.3 Z24^−^^/^^−^ Mice Have Increased Myelopoiesis and Diminished Lymphopoiesis 3 Days Following Fracture

**Figure 3:**
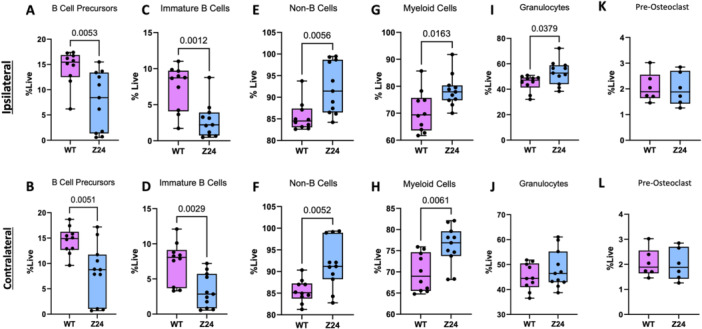
Differential B cell development, myeloid and neutrophil populations in Z24^−/−^ bone marrow 3 days post‐fracture. Spectral flow immunophenotyping analysis of ipsilateral and contralateral tibia bone marrow collected 3 days post‐fracture evaluating the quantity of live (A–B) B cell precursors, (C–D) immature B cells, (E–F) non–B cells, (G–H) myeloid cells, (I–J) neutrophils, and (K–L) pre‐osteoclasts. (*n:* WT = 10, Z24^−^^/^^−^ = 11). *p* ≤ 0.05 determined by two‐tailed T‐test.

**Figure 4:**
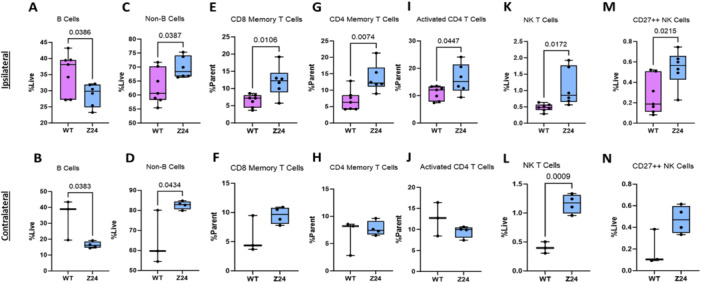
Differential B cell population, T cell activation, and natural killer cell repertoire in Z24^−^^/^^−^ ipsilateral lymph nodes. Spectral flow immunophenotyping analysis of ipsilateral and contralateral inguinal lymph nodes collected 3 days post‐fracture evaluating the quantity of (A–B) B cells, (C–D) non‐B cells, (E–F) CD8^+^ memory T cells, (G–H) CD4^+^ memory T cells, (I–J) activated CD4^+^ T cells, (K–L) NK T cells, and (M–N) CD27^++^ NK cells. (*n*: WT = 3–7, Z24^−^^**/**^^−^ = 4–6). *p* ≤ 0.05 determined by two‐tailed T‐test.

Based on the observed differences in the circulating PBMCs, we next completed detailed immunophenotyping of the ipsilateral (fractured tibia) and contralateral (unfractured tibia) bone marrow compartments. (Figures 3, 4 **)** While all time points were analyzed (data not shown), the most significant differences were observed during the pro‐inflammatory phase of fracture healing 3 days post‐fracture. Z24^−^^/^^−^ mice displayed significant evidence of diminished lymphopoiesis in the bone marrow on both the ipsilateral and contralateral sides 3‐days after fracture. This is evidenced by decreased B cell precursors (*p* = 0.0053, *p* = 0.0051, respectively; Figure 3A–B), decreased immature B cells (*p* = 0.0012, *p* = 0.0029, respectively; Figure 3C–D), and increased quantity of non‐B cells (*p* = 0.0056, *p* = 0.0052, respectively; Figure 3E–F).

Conversely, Z24^−^^/^^−^ mice presented with increased myeloid cells in both the ipsilateral (*p* = 0.0163) and contralateral (*p* = 0.0061) limbs (Figure 3G–H). Neutrophils were only significantly increased in the ipsilateral limb (*p* = 0.0379, Figure 3I–J). Neither the macrophage (Supporting Figure 2E) or pre‐osteoclast (*p* = 0.2577 ipsilateral and *p* = 0.8697 contralateral, Figure 3K–L) populations were significantly different in the bone marrow of the Z24^−^^/^^−^ mice compared to WT mice.

Z24^−^^/^^−^ mice again displayed signs of diminished quantity of B cells on both the ipsilateral and contralateral sides in the inguinal lymph nodes, evidenced by decreased B cell populations (*p* = 0.0386 Figure 4A; *p* = 0.0383, Figure 4B) and increased quantity of non‐B cells (*p* = 0.0387, Figure 4C; *p* = 0.0434, Figure 4D). There were also significant changes to the T cell populations with increased CD4^+^ (*p* = 0.0074, Figure 4G) and CD8^+^ memory T cells (*p* = 0.0106, Figure 4E), and activated CD25^+^CD4^+^ T helper cells (*p* = 0.0447, Figure 4I) in Z24^−^^/^^−^ mice relative to WT mice. This hyperactivated T cell response is only present in the ipsilateral lymph nodes (Figure 4F,H,J). Z24^−^^/^^−^ mice also presented with significantly increased numbers of natural killer (NK) T cells on both the ipsilateral (*p* = 0.0172, Figure 4K) and contralateral (*p* = 0.0009, Figure 4L) lymph nodes and increased CD27^++^ NK cells (*p* = 0.0215, Figure 4G) in the ipsilateral lymph node.

### 3.4 Z24^−^^/^^−^ Mice Phenocopy the Delayed Fracture Healing Phenotype of Aged Mice With Frail Bones and Cellular Senescence

**Figure 5:**
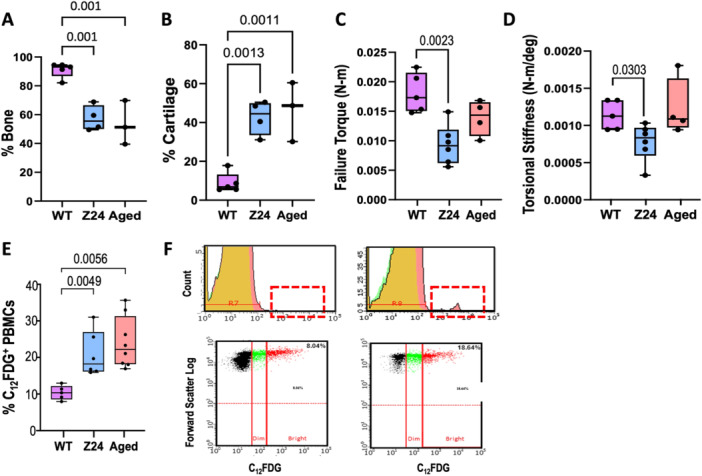
Delayed fracture healing, fragility and cellular senescence in Z24^−^^/^^−^ mice. (A) Histomorphometric quantification of bone and (B) cartilage composition in tibial fracture callus 21 days post‐fracture (*n*: WT = 5, Z24^−/−^ = 4, Aged WT = 3). (C) Maximum torque withstood by unfractured mice tibiae. (D) Torsional stiffness of mice tibiae calculated from failure torque and angle of twist (*n*: WT = 5, Z24 = 6, Aged = 4). (E) Z24 and Aged WT mice have higher C12FDG PBMC senescence 3‐days following (*n*: WT = 5, Z24 = 6, Aged = 8). (F) Representative flow cytometry readouts for WT (left) and Z24 (right) mice PBMCs collected after fracture and stained with C12FDG. The “bright” cell population (red) is considered senescent. Significance was defined by *p *≤ 0.05 determined by one way ANOVA followed by Tukey's post‐hoc testing.

At 21 days post‐fracture, histomorphometry demonstrated delayed facture healing in both the Z24^−^^/^^−^ and aged WT relative to the 12‐week old WT mice. Bone percentage was significantly higher in WT relative to either Z24^−^^/^^−^ or aged WT, with no significant difference between Z24^−^^/^^−^ and aged WT (WT 91.12%, Z24^−^^/−^ 57.37%, aged WT 53.56%; Figure 5A). Conversely, there was significantly more retained cartilage in both Z24^−^^/^^−^ and aged WT relative to WT, with no significant difference between Z24^−^^/^^−^ and aged WT (WT 8.90%, Z24^−^^/^^−^ 42.63%, aged WT 46.44; Figure 5B). Mechanical integrity of the unfractured, contralateral tibiae of Z24^−^^/^^−^ mice demonstrated average failure torque and torsional stiffness that were 48.0% (*p* = 0.0087, Figures 5C) and 31.9% (*p* = 0.0303, Figure 5D) lower than WT mice, respectively. Aged mice did not differ significantly from either group. Cellular senescence in the Z24^−^^/^^−^ and aged WT was significantly higher in the peripheral blood mononuclear cells 3‐days following fracture relative to WT (WT 10.35%, Z24^−^^/^^−^ 20.87%, aged WT 24.08%; Figure 5E–F). However, we did not find a significant difference in the local expression of the cell cycle genes commonly associated with senescence, p16 (Supporting Figure 3A) as and p21 (Supporting Figure 3B), in the Z24^−^^/^^−^ relative to WT.

**Figure 6:**
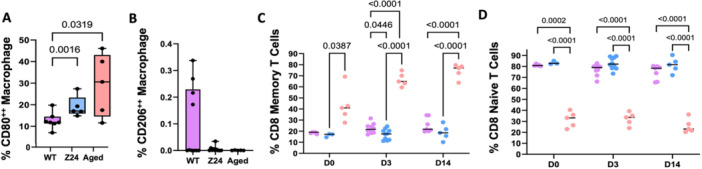
Immune dysregulation in Z24^−/−^ and aged wild‐type mice. (A–B) Immunophenotyping analysis of peripheral blood at day 3 post‐fracture evaluating the quantity of pro‐inflammatory M1 CD80+ and pro‐regenerative M2 CD206+ macrophages (*n*: WT = 10, Z24^−/−^ = 10, Aged WT = 5). Significance was defined by *p* ≤ 0.05 determined by one‐way ANOVA followed by Tukey's post‐hoc testing. Time course analyses of peripheral blood collected before fracture and 3‐ and 14 days post‐fracture evaluating the quantity of (C) CD8+ memory T cells and (D) CD8+ naïve T cells. (*n*: WT = 5–10, Z24^−/−^ = 3–10, Aged WT = 5). *p* < 0.05 determined by mixed‐effects analysis (two‐way ANOVA) with Tukey multiple comparisons correction test.

The Z24^−^^/^^−^ and aged WT showed consistent macrophage dysregulation with significantly higher pro‐inflammatory M1 CD80^++^ macrophages (Figure 6A) and lower pro‐regenerative M2 CD206^++^, although this difference was not significant due to the large variance in the WT mice (Figure 6B). Within the fracture callus there was mixed evidence of an altered local pro‐inflammatory microenvironment with a significantly increased expression of *Tnf‐α* in the Z24^−^^/^^−^ mice (Supporting Figure 3C, *p* = 0.0303) but not *Il‐1β* (Supporting Figure 3D **)** as measured by qRT‐PCR. However, T cells in the peripheral blood of Z24^−^^/^^−^ and aged WT mice diverged with the aged mice having a significantly increased number of CD8^+^ memory T cells (Figure 6C) and decreased naïve CD8^+^ T cells (Figure 6D) in the aged mice relative to either the Z24^−^^/^^−^ or WT mice.

Multiplex and ELISA analysis of plasma 21 days post‐fracture was performed to evaluate systemic differences in a subset of proteins established in the context of aging, senescence, inflammation, and bone healing (eotaxin, IP‐10, GDF‐15, IL‐13) [8, 33]. Z24^−^^/^^−^ mice demonstrated no significant difference in eotaxin, IP‐10, and GDF‐15 compared to the age‐match WT mice (Supporting Figure 4A–C). However, there was a > 2‐fold higher level of IL‐13 in Z24^−^^/^^−^ mice compared to age‐matched WT or aged WT mice (Supporting Figure 4D). Eotaxin and GDF‐15 were significantly higher in the aged WT mice (Supporting Figure 4A,C).

## 4 Discussion

Several studies have utilized the Z24^−^^/^^−^ progeria mouse to model aging‐associated musculoskeletal conditions and frailty [19, 20]. In the present study, our primary objective was to validate Z24^−^^/^^−^ as a small‐animal model of delayed fracture healing relative to WT. Multiple studies have established that by 3 months of age Z24^−^^/^^−^ mice present with an osteoporotic/osteopenic phenotype with decreased bone mineral density, loss of trabecular and cortical bone, and increased trabecular spacing, along with reports of lower weight, loss of hair, and a malnourished appearance [34, 35, 36, 37]. This accelerated aging phenotype is caused by a mutation in the Z24 metalloproteinase that leads to nuclear blebbing and cellular stiffness due to the disrupted assembly of lamin in the cytoskeleton, a process that also occurs during natural aging [34, 38, 39, 40, 41]. It has also been demonstrated that reduced lamin A/C [42] can lead to impaired osteogenesis with increased adipogenesis [43, 44, 45], and that frail individuals have reduced circulating osteoprogenitors [22].

Our research is the first to explore the Z24^−^^/^^−^ mouse model in the context of fracture healing. General skeletal frailty in Z24^−^^/^^−^ mice at the time of fracture (10–14 weeks of age) was confirmed through the decreased failure torque and torsional stiffness in the tibiae of the Z24^−^^/^^−^ mice. Consistent with murine experiments demonstrating delayed endochondral conversion of cartilage to bone in naturally aged mice [13, 14, 46], Z24^−^^/^^−^ mice presented with retained cartilage and decreased bone fractions in the fracture callus compared to age‐matched young WT mice. While we cannot fully eliminate a possible defect in the osteoclast activity remodeling the cartilage, day 3 following fracture the pre‐osteoclast population was not significantly different between the Z24^−^^/^^−^ and WT mice. The Z24^−^^/^^−^ mice also developed a smaller overall fracture callus, similar to other murine models of impaired fracture healing [47, 48, 49]. As previously proposed, this may be due to the depletion of the functional periosteal stem cell progenitor population [13, 48].

Immune dysregulation with natural aging, commonly referred to as “inflammaging” [50], is causal in age‐related morbidities of solid organs [51, 52] and delayed fracture repair [14, 46]. To characterize differences in the immunophenotype between the Z24^−^^/^^−^ mice and age‐matched WT mice, we completed spectral flow cytometry on bone marrow, lymph nodes, and peripheral blood to identify a broad spectrum of the major myeloid and lymphoid cell populations. The systemic immune response was analyzed by immunophenotyping of the peripheral blood and compared to the local immune response within the bone marrow and inguinal lymph node that drains the tibial fracture site. Significant differences in both the innate and adaptive immune systems were observed between the two mice cohorts. Within the innate response, 3 days after fracture, we saw significantly more neutrophils and myeloid cells within the bone marrow of Z24^−^^/^^−^ mice and fewer circulating anti‐inflammatory macrophages. Neutrophils are the most abundant cells recruited to the fracture site in the inflammatory stage and release damage associated molecular patterns (DAMPs) that are critical to initiating the endogenous innate immune response but are associated with poor outcomes when present in excess. Supporting this, preclinical neutrophil depletion studies demonstrate they are essential to the normal healing process [53], but most clinical studies show that higher neutrophil concentrations are associated with an increased risk of severe complications, delayed union, and even mortality in geriatric patients (as recently reviewed [54]). As a consequence, in the Z24^−^^/^^−^ mice we measure a sustained upregulation of the local pro‐inflammatory microenvironment with significantly increased expression of *tnf‐α* within the fracture callus compared to WT mice even as far out as 9 days post‐fracture. Our data coupled with prior studies suggest that the higher concentration of neutrophils in the Z24^−^^/^^−^ mice may contribute to the pro‐inflammatory micro‐environment and delayed healing phenotype that we observed.

The increased concentration of neutrophils was concomitant with an increase in myeloid cells and a decrease in B lymphocytes, which are hallmark immune changes observed with natural aging [55, 56]. While we did see a higher number of total myeloid cells in the bone marrow, we measured a significant reduction in the number of anti‐inflammatory CD163^+^ and CD206^+^ circulating macrophages 3 days following fracture, with these lower quantities generally being maintained throughout the time course of healing. We further saw an increased number of circulating pro‐inflammatory CD80^++^ macrophages in the Z24^−^^/^^−^, consistent with the aged WT mice.

Significant adaptive immune system changes were also observed systemically and locally in Z24^−^^/^^−^ mice relative to age‐matched WT mice. In the bone marrow and draining lymph nodes the, Z24^−^^/^^−^ mice demonstrated a dramatic reduction in the number of B cells. This finding is consistent with literature showing that a reduction in lymphopoiesis is a hallmark of aging [55]. Moreover, an infiltration of B cells is one of the strongest positive predictors of optimal bone healing [33], supporting our correlation between the poor fracture healing in the Z24^−^^/^^−^ mice and decreased B‐cells in the draining lymph node.

The T cell phenotype was more difficult to equate to existing literature due to the diversity in T cell labeling strategy used across the various flow cytometry protocols. In our study we found Z24^−^^/^^−^ mice had higher numbers of naïve CD8^+^ T cells and reduced differentiated cytotoxic CD8^+^ T cells (both memory and activated) in the blood but an accumulation of cytotoxic cells within the lymph node relative to age‐matched WT mice. We also found higher number of CD4^+^ T‐helper cells systemically in the blood and within the lymph node of Z24^−^^/^^−^ compared to the age‐matched. Notably, the increase in Z24^−^^/^^−^ CD4^+^ and CD8+ memory cells was only present in the fracture side (ipsilateral) lymph node, not in the contralateral lymph node or systemically in the blood. However, naturally aged mice presented with higher CD8^+^ memory cells and fewer naïve CD8^+^ T cells systemically compared to either Z24^−^^/^^−^ or adult WT. Previously published work has found that an increase in CD8^+^ effector memory T cells (CD3^+^CD8^+^CD11a^++^CD28^−^ CD57^+^) was highly correlated to delayed fracture healing [57]. The complex T cell changes suggest that perhaps systemic changes in adaptive immune response of the young progeria mice has not fully recapitulated the aged WT mice, but that the increase in Z24^−^^/^^−^ myeloid cells found in the bone marrow may promote a hyperactivation of T cells local to the fracture side in the progeria mice.

Interestingly, we also found a significant increase in CD27^++^ NK and NK T cells in the Z24^−^^/^^−^ mice relative to age‐matched WT. To our knowledge, there is no comparative research currently available on these cell types during fracture healing, and therefore, these cells represent an interesting avenue for future research. In general, the role of NK cells in trauma is not well understood, and a recent review of the literature suggests that there is a tight interplay between NK cells and mesenchymal stromal cells during fracture healing [54].

We complemented the immunophenotyping assessment with quantification of cellular senescence. The most widely used biomarker of cellular senescence is increased activity of the acidic senescence‐associated β‐galactosidase (SA‐β‐gal) [58]. C_12_FDG is a compound that fluoresces at 514 nm when hydrolyzed by intracellular SA‐β‐gal and has been well described for use in mice [31, 32] and, more recently, our work in humans [59]. Using this technique, we found that Z24^−^^/^^−^ progeria mice and aged WT mice have significantly increased number of systemic C_12_FDG^+^ PBMCs before fracture compared to the adult WT mice, with an even further relative accumulation of these cells 3‐days following fracture. We also found an upregulation of local *Tnf‐α* expression within the fracture callus, a pro‐inflammatory marker widely considered to be a canonical SASP (senescent associated secretory phenotype) [60, 61]. However, we did not see a local increase in the expression of *p16* or *p21*, cell cycle arrest programs commonly associated with cellular senescence [60, 61], within the fracture callus 9 days post fracture. These markers alone may be imperfect markers of a true cellular senescence in the context of fracture repair as emerging data suggest that there is a cell‐specific and temporal role for these markers in repair [62, 63]. Future studies taking a multiparametric approach to contrasting systemic versus local cellular senescence will add further insight into how cellular senescence in Z24^−^^/^^−^ progeria mice compares to natural aging [57, 64, 65].

We also analyzed a limited number of candidate cytokines associated with the inflammatory and senescent associated secretory phenotype for differential systemic expression 21 days post‐fracture based on prior work suggesting predictive relationships with bone regeneration after trauma [33]. In our model, we found very few of these cytokines had differences in expression, with the exception of IL‐13, which was significantly upregulated in Z24^−^^/^^−^ mice fracture compared to the WT mice. IL‐13 has a high degree of structural homology with IL‐4 and is commonly considered an anti‐inflammatory cytokine. In fracture healing, early upregulation of IL‐13 was recently identified as a biomarker of successful healing in femoral defects with delayed treatment in otherwise healthy young adult‐age rats [33]. However, IL‐13 is also associated with chronic inflammatory diseases leading to fibrosis, systemic sclerosis, and asthma [66, 67]. IL‐13 is also made by NK and activated T helper cells, both of which were found to be higher in the draining lymph node, but not the contralateral lymph node, of Z24^−^^/^^−^ mice compared to WT [67]. Consequently, in this Z24^−^^/^^−^ model of accelerated aging, the increased IL‐13 at the late stages of healing where we measured expression, may instead be associated with a dysregulated immune state and contribute to the increased fibrosis observed in the fracture callus of the Z24^−^^/^^−^ mice.

The Z24^−^^/^^−^ model has some noted limitations. The pathology of bone fracture healing is complex and the different phases of fracture repair present shifting biological environments that may not have been equivalent in the Z24^−^^/^^−^ and WT littermate mice due to the “accelerated” timescale of aging with the progeria phenotype. Furthermore, the process of aging in general is not universal, evidenced by some elderly animals presenting as “super‐agers” with immunological states that align more closely with young mice [14]. Similarly, we found more variance in the quantitative fracture healing and immunosenescent outcomes of Z24^−^^/^^−^ mice compared to the age‐matched WT mice.

The Z24^−^^/^^−^ mice are also smaller than their age‐matched WT counterparts with a frail behavioral phenotype that we, and others, have also shown are associated with osteoporotic and sarcopenic standards. This contrasts naturally aged mice from controlled breeding settings which tend to be heavier than young adult mice and their bone strength as not significantly diminished. These different weight spectrums between Z24^−^^/^^−^, young adult WT, and naturally aged WT mice may also influence healing outcomes as there is an interconnected nature between bone loading and fracture repair. However, the frailty, sarcopenia, and osteoporosis of the Z24^−^^/^^−^ mice aligns more closely with the characteristics of aged humans. Including behavioral and kinematic analyses of functional fracture healing in the future could help parse out how factors such as weightbearing or movement contributed the repair, or if this process segregates according to age or genotype as has recently been investigated according to fracture fixation and sex [27].

Lastly, we also acknowledge that due to the limitation of Mendelian frequency of the Z24^−^^/^^−^ mice from the Z24^+/^^−^ parents, we were unable to segregate our data set by mouse sex due to an unequal distribution of male to female mice. The inclusion of both sexes may have contributed to increased deviation across some of our analyses. For example, in Figure 2B the four high measurements were only within female mice; however, if male mice were excluded from the analysis the statistical outcome was not changed. While our previous work has not found significant differences in fracture healing according to sex in adult WT mice [25, 26, 27], future molecular or cellular profiling of the Z24^−^^/^^−^ could be more profound through the segregation of sex, since sex‐differences have recently been demonstrated in naturally aged mice [68].

Despite these limitations, here we present Z24^−^^/^^−^ mice as a murine model of delayed healing that has many parallels to aging associated co‐morbidities that drive delayed fracture healing in human populations and natural aging in mice. There has been a gap in models that enabled testing of therapeutic approaches to accelerate fracture healing in situations of delayed bone repair that translates well to clinical experiences. Because the complex mechanics and biology of delayed and nonunion fractures remain poorly understood, management of these injuries poses a significant challenge to clinicians in deciding to either wait for healing to occur or intervene with either surgical or non‐surgical methods. The fracture healing phenotype of the Z24^−^^/^^−^ mouse represents a new preclinical translational tool that is more feasible than natural aging to test efficacy and mechanism of action for novel therapeutic strategies designed to accelerate bone healing in aged individuals.

## Author Contributions

**Victoria R. Duke:** Formal Analysis, Investigation, Data Curation, Writing – Original Draft, Writing – Review and Editing, Visualization. **Marc J. Philippon Jr.:** Formal Analysis, Investigation, Data Curation, Writing – Original Draft, Writing – Review and Editing, Visualization. **Dane R.G. Lind:** Formal Analysis, Investigation, Data Curation, Writing – Original Draft, Writing – Review and Editing, Visualization. **Herbert Kasler:** Methodology, Formal Analysis, Investigation, Data Curation, Resources, Writing – Review and Editing. **Kohei Yamaura:** Investigation. **Matt Huard:** Investigation**. Molly Czachor:** Investigation, Writing – Review and Editing. **Justin Hollenbeck:** Investigation. **Jacob D. Matityahu:** Investigation; Writing – Review and Editing **Justin Brown:** Investigation. **Alex Garcia:** Investigation. **Naomasa Fukase:** Investigation. **Ralph S. Marcucio:** Methodology, Validation, Writing – Review and Editing. **Anna‐Laura Nelson:** Investigation, Writing – Review and Editing. **William S. Hambright:** Conceptualization, Methodology, Validation, Supervision, Writing – Review and Editing. **Dustin M. Snapper:** Formal Analysis, Investigation, Writing – Original Draft, Writing – Review and Editing. **Johnny Huard:** Conceptualization, Methodology, Resources, Funding Acquisition, Writing – Review and Editing. **Chelsea S. Bahney:** Conceptualization, Methodology, Resources, Funding Acquisition, Supervision, Formal Analysis, Writing – Original Draft, Writing – Review and Editing.

## Conflicts of Interest

Dr. Bahney discloses IP royalties from Iota Biosciences Inc. for US Patent 041263 and an Associate Editor role for the Journal of Tissue Engineering and Regenerative Medicine (JTERM).

## Supporting information


**Figure S1:** Immunophenotyping gating strategy. **Figure S2:** Spectral flow immunophenotyping analysis of peripheral blood (A‐B) and bone marrow (C‐E) for cell types with non‐significant differences between WT and Z24 mice. **Figure S3:** Quantitative RT‐PCR of fracture callus 9‐days following fracture for senescent‐related genes (A) p16 and (B) p21 or senescence‐associated secretory phenotype (SASP) genes (C) Tnf‐a and (C) Il‐1b. (n: WT=5, Z24=6). **Figure S4:** Multiplex and ELISA protein analysis of serum collected at 21 days post‐fracture. **Table S1:** Antibodies for immunophenotyping.
