# Supplementary material for: Murine Progeria Model Exhibits Delayed Fracture Healing With Senescent Phenotype and Dysregulated Immune Response
Source: J Orthop Res. 2026 Apr 4;44(4):e70193. doi: 10.1002/jor.70193 (PMC13049502; doi:10.1002/jor.70193)
Supplement: Supplementary file 1 — Figure S1: Immunophenotyping gating strategy. Figure S2: Spectral flow immunophenotyping analysis of peripheral blood (A‐B) and bone marrow (C‐E) for cell types with non‐significant differences between WT and Z24 mice. Figure S3: Quantitative RT‐PCR of fracture callus 9‐days following fracture for senescent‐related genes (A) p16 and (B) p21 or senescence‐associated secretory phenotype (SASP) genes (C) Tnf‐a and (C) Il‐1b. (n: WT=5, Z24=6). Figure S4: Multiplex and ELISA protein analysis of serum collected at 21 days post‐fracture. Table S1: Antibodies for immunophenotyping. [file JOR-44-0-s001.docx]

**SUPPLEMENTARY**

**Supplementary Figure 1. Immunophenotyping Gating Strategy Example**

**A**


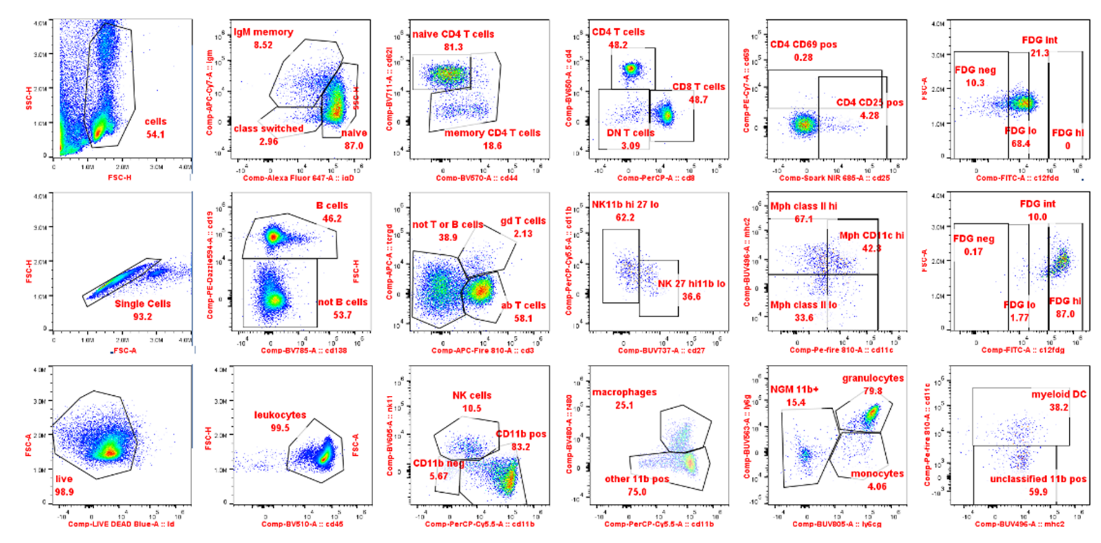


**B**


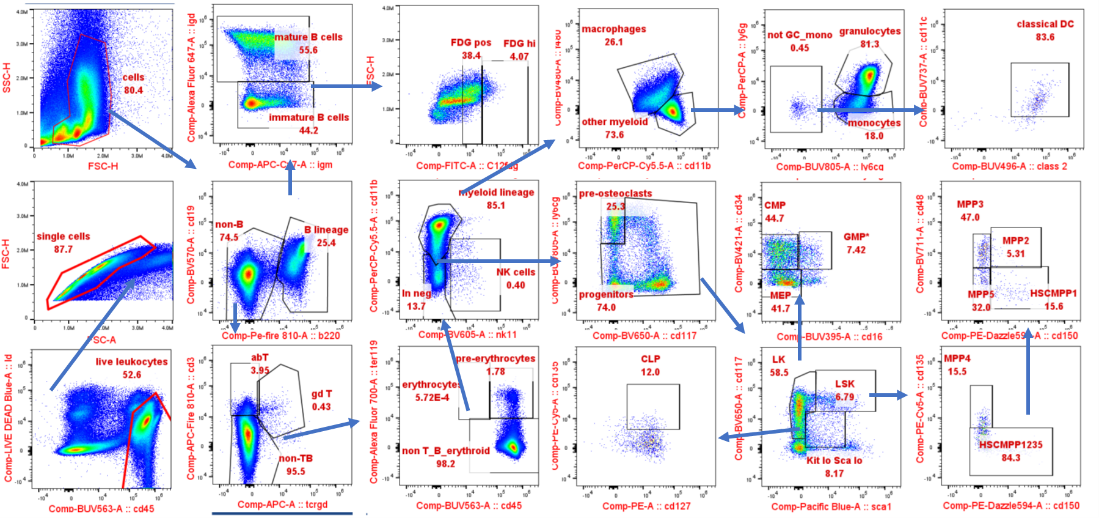


***Supplementary Figure 1. Immunophenotyping gating strategy.*** *(A) Representative gating with 30-color panel for main populations in blood and tissues. (B) Representative gating with 30-color panel for main populations in bone marrow.*

***Supplemental Figure 2.*** *Spectral flow immunophenotyping analysis of peripheral blood* ***(A-B)*** *and bone marrow* ***(C-E)*** *for cell types with non-significant differences between WT and Z24 mice.*

***Supplemental Figure 3.*** *Quantitative RT-PCR of fracture callus 9-days following fracture for senescent-related genes* ***(A)*** *p16* and ***(B)*** *p21* or senescence-associated secretory phenotype (SASP) genes ***(C)*** *Tnf-α and* ***(C)*** *Il-1β. (n: WT=5, Z24=6).*

******

***Supplemental Figure 4. Multiplex and ELISA protein analysis of serum collected at 21 days post-fracture.*** ***(A)*** *Eotaxin levels were increased in Aged WT mice compared to WT and Z24 mice (n: WT=3, Z24=6, AgedWT=8).* ***(B)*** *IP-10 levels were lowest in Z24 mice, although this difference was not significant (n: WT=3, Z24=7, AgedWT=7).* ***(C)*** *GDF-15 levels were significantly higher in Aged WT mice compared to Z24 mice (n: WT=5, Z24=9, AgedWT=9).* ***(C)*** *IL-13 levels were significantly higher in Z24 than WT and Aged WT mice (n: WT=3, Z24=8, AgedWT=4). One way ANOVA followed by Tukey’s post hoc testing used to determine significance.*

**Supplementary Table 1.** Antibodies for immunophenotyping.

| **Target** | **Fluorochrome** | **Clone** | **Manufacturer** | **Cat #** | **Volume per sample (µL)** |
| --- | --- | --- | --- | --- | --- |
| Ter-119 | AF700 | TER-119 | BioLegend | 116220 | 0.50 |
| IgD | Alexa_Fluor_647 | 11-26c.2a | BioLegend | 405707 | 0.125 |
| tcrgd | APC | GL3 | BioLegend | 118115 | 0.25 |
| IgM | APC-Cy7 | RMM-1 | BioLegend | 406531 | 0.50 |
| CD3 | APC-Fire_810 | 17A2 | BioLegend | 100267 | 0.50 |
| CD16 | BUV396 | 190909 | BD Biosciences | 747953 | 1.25 |
| Class II | BUV496 | M5/114.15.2 | BD Biosciences | 750281 | 0.30 |
| CD45 | BUV563 | 30-F11 | BD Biosciences | 612924 | 0.50 |
| CD11c | BUV737 | HL3 | BD Biosciences | 612796 | 0.50 |
| Ly-6C | BUV805 | HK1.4.rMAb | BD Biosciences | 755202 | 0.50 |
| CD34 | BV421 | MEC14.7 | BioLegend | 119321 | 0.50 |
| F4/80 | BV480 | T45-2342 | BD Biosciences | 565635 | 0.50 |
| CD41 | BV510 | MWReg30 | BioLegend | 133923 | 0.25 |
| CD19 | BV570 | 6D5 | BioLegend | 115535 | 0.50 |
| NK1.1 | BV605 | PK136 | BioLegend | 108739 | 0.50 |
| CD117 | BV650 | 2B8 | BioLegend | 105853 | 0.25 |
| CD48 | BV711 | HM48-1 | BioLegend | 103439 | 0.125 |
| CD38 | BV785 | 90/CD38 | BD Biosciences | 740887 | 0.50 |
| Sca-1 | Pacific Blue | D7 | BioLegend | 108119 | 0.50 |
| CD127 | PE | S18006K | BioLegend | 158203 | 0.25 |
| CD135 | PE-Cy5 | A2F10 | BioLegend | 135311 | 0.50 |
| CD80 | PE-Cy7 | 16-10A1 | BioLegend | 104733 | 0.125 |
| CD150 | PE-Dazzle_594 | TC15-12F12.2 | BioLegend | 115935 | 0.125 |
| B220 | PE-Fire_810 | RA3-6B2 | BioLegend | 103287 | 0.125 |
| CD206 | PE-Fire700 | C068C2 | BioLegend | 141741 | 0.20 |
| LY-6G | PerCP | 1A8 | BioLegend | 127653 | 1.00 |
| CD11b | PerCP-Cy5.5 | M1/70 | BioLegend | 101227 | 0.25 |
| CD163 | PerCP-eFluor_710 | TNKUPJ | ThermoFisher | 46-1631-80 | 0.50 |

**Supplementary Table 2.**
